# Nuclear localization of orphan receptor protein kinase (Ror1) is mediated through the juxtamembrane domain

**DOI:** 10.1186/1471-2121-11-48

**Published:** 2010-06-30

**Authors:** Hsiao-Chun Tseng, Ping-Chiang Lyu, Wen-chang Lin

**Affiliations:** 1 Institute of Bioinformatics and Structural Biology, College of Life Science, National Tsing Hua University, Hsinchu 300, Taiwan; 2 Institute of Biomedical Sciences, Academia Sinica, Taipei 115, Taiwan

## Abstract

**Background:**

Several receptor tyrosine kinases (RTKs) such as EGFR, FGFR, TRK, and VEGFR are capable of localizing in the cell nucleus in addition to their usual plasma membrane localization. Recent reports also demonstrate that nuclear-localized RTKs have important cellular functions such as transcriptional activation. On the basis of preliminary bioinformatic analysis, additional RTKs, including receptor tyrosine kinase-like orphan receptor 1 (Ror1) were predicted to have the potential for nuclear subcellular localization. Ror1 is a receptor protein tyrosine kinase that modulates neurite growth in the central nervous system. Because the nuclear localization capability of the Ror1 cytoplasmic domain has not been reported, we examined the cellular expression distribution of this region.

**Results:**

The Ror1 cytoplasmic region was amplified and cloned into reporter constructs with fluorescent tags. Following transfection, the nuclear distribution patterns of transiently expressed fusion proteins were observed. Serial deletion constructs were then used to map the juxtamembrane domain of Ror1 (aa_471-513) for this nuclear translocation activity. Further site-directed mutagenesis suggested that a KxxK-16 aa-KxxK sequence at residues 486-509 is responsible for the nuclear translocation interaction. Subsequent immunofluorescence analysis by cotransfection of Ran and Ror1 implied that the nuclear translocation event of Ror1 might be mediated through the Ran pathway.

**Conclusions:**

We have predicted several RTKs that contain the nuclear localization signals. This is the first report to suggest that the juxtamembrane domain of the Ror1 cytoplasmic region mediates the translocation event. Ran GTPase is also implicated in this event. Our study might be beneficial in future research to understand the Ror1 biological signaling pathway.

## Background

Receptor tyrosine kinases (RTKs) are transmembrane molecules situated at the cellular surface whose function is to detect their specific cognate ligands in the extracellular milieu. RTKs are critical signal-transduction mediators that regulate many essential cellular activities including growth and differentiation. In the usual signal-transduction pathways, RTKs need to relay in a stepwise manner the signals from cellular surface through other signaling molecules, such as nonreceptor tyrosine kinases and serine/threonine kinases. In addition to the anticipated cellular surface localization of RTKs, intriguingly, recent reports have indicated that some of these receptor kinases can be translocated inside the nucleus and may constitute newly identified biochemical signals by themselves [1-3]. These RTKs include the EGFR family (EGFR [4,5], ErbB-2 [6,7], ErbB-3 [8], ErbB-4 [9-11]), FGFR family (FGFR1 [12-14], FGFR3 [15]), TRKA [16,17], and VEGFR2 [18,19].

Although the details of the biological functions of these nuclear translocated RTKs are not understood fully, studies have indicated the unique biochemical roles of RTKs when trafficked into the nucleus. Membrane RTKs are known to participate in the signal transduction pathways including the Ras-MAPK pathway, IP3/DAG pathway, and PI3K pathway [20,21]. By contrast, nuclear ErbBs can function as transcription modulators in addition to the protein kinase signal transducer [22]. For example, nuclear EGFR interacts with STAT5 or STAT3 to transactivate the expression of the Aurora-A or nitric oxide synthase (iNOS) genes, respectively [5,23]. Nuclear EGFR can also function as a protein tyrosine kinase to phosphorylate Tyr 211 of PCNA, which increases the stability of chromatin-bound PCNA protein [24]. Several recent studies indicate that the nuclear localization of the ErbB proteins can be a pathological feature in tumors such as breast carcinoma, oropharyngeal squamous cell carcinoma, and ovarian cancer tumors [25-29].

Another study of RTKs showed that activation of the tyrosine hydroxylase gene promoter by nuclear FGFR1 and its natural ligand (FGF-2) is mediated through the cAMP-responsive element (CRE) complexes in addition to the usual intermediate signaling kinase molecules [13]. Learning more about the RTK nuclear localization process and the underlying mechanisms would lead to a better understanding of the modulatory functions of RTKs in cells. In this study, we used bioinformatic tools to scan all known human RTKs for their potential to localize in the nucleus, and we conducted molecular experiments to dissect the nuclear localization domain of one RTK, receptor tyrosine kinase-like orphan receptor 1 (Ror1).

In our preliminary analysis, we first predicted the potential RTK protein subcellular locations using the PSORT II program. Our results indicated that several additional RTKs might also be capable of localizing in the nucleus, including the ROR RTK family. The ROR family comprises two structurally related RTK genes, Ror1 and Ror2, which share an overall 58% amino acid identity. Rors were first cloned from a neuroblastoma cell line using a polymerase chain reaction (PCR)-based approach, which showed a region that is strongly homologous to the tyrosine kinase domain of the Trk family [30]. Rors are evolutionally conserved among *Caenorhabditis elegans*, *Aplysia *[31], *Drosophila melanogaster *[32], mice [33], rats, cows, dogs, chimpanzees, and humans. The ligand of Ror2 is Wnt-5A [34], whereas Ror1 remains an orphan receptor protein tyrosine kinase without any known interacting ligand molecules.

Ror1 and Ror2 are newly identified receptor tyrosine kinases to have function involved in development in mammalian central neurons. The Rors protein accumulates in the process of synapse formation and concentrated in the growth cone of the immature neuron [35]. In addition, knockdown of Ror1 or Ror2 expression leads to a shorter and less branched neurite extension phenomenon [36]. Some reports suggest that Ror1 and Ror2 have different subcellular localizations, respectively. Ror1 associates with F-actin microfilament, whereas Ror2 co-localizes with microtubules [37]. More studies are needed to reveal the biological functions of ROR RTKs. Here, we report on the confirmation of the nuclear localization potential of this RTK family and our characterization of the critical region involved in the nuclear translocation of Ror1.

## Results

### Prediction of nuclear-localized RTKs

To learn more about the nuclear distribution of RTKs in cells, we used the PSORT II program to predict the nuclear localization probabilities of all human RTKs. The PSORT II program was developed by Dr. Nakai's group (Human Genome Center, Institute for Medical Science, University of Tokyo, Japan) using the *k*-nearest-neighbor method. The *k*-nearest-neighbor method is a pattern-recognition algorithm in which *k *is a predefined parameter. The percentage of proteins whose nuclear localization sites are correctly predicted is up to 90.7% [38]. Protein subcellular prediction by PSORT II indicated that several RTKs could localize in the nucleus. Table 1 shows the predicted nuclear-localized RTKs including the EGFR, FGFR, ROR, VEGFR, AATYK, and TRK RTK families. Some reports have demonstrated that members of the EGFR, FGFR, VEGFR, and TRK families localize to the nucleus [4-19].

**Table 1:** Summary of nuclear-localized RTKs by PSORT II prediction

	PSORT II results (NNCN score)	References
**EGFR family**: EGFR	Nuclear (70.6)	Dittmann et al., 2005 [46]; Dittmann et al., 2008 [47]; Hoshino et al., 2007 [48]; Hsu and Hung, 2007 [4]; Hu et al., 2007 [49]; Hung et al., 2008 [5]; Lo et al., 2006a [50]; Lo et al., 2005 [23]; Lo et al., 2006b [51]; Lo and Hung, 2007 [52]

ERBB-2	Cytoplasmic (55.5)	Xie and Hung, 1994 [6]; Giri et al., 2005 [7]; Wang et al., 2004 [53]

ERBB-3	Nuclear (70.6)	Cheng et al., 2007 [8]

ERBB-4	Nuclear (55.5)	Feng et al., 2009 [11]; Ni et al., 2003 [9]; Sundvall et al., 2007 [54]; Williams et al., 2004 [10]

**FGFR family**: FGFR1	Nuclear (55.5)	Kilkenny and Hill, 1996 [12]; Myers et al., 2003 [14]; Peng et al., 2002 [13]; Stachowiak et al., 1996 [55]

FGFR3	Cytoplasmic (89)	Johnston et al., 1995 [15]

**ROR family**: ROR1	Nuclear (89)	None reported

ROR2	Nuclear (70.6)	None reported

**VEGFR family**: VEGFR1	Nuclear (70.6)	None reported

VEGFR2	Cytoplasmic (76.7)	Pillai et al., 2005 [18]; Zhang et al., 2005 [19]

**AATYK family**: LMTK2	Nuclear (89)	None reported

**TRK family**: NTRK1	Cytoplasmic (94.1)	Bonacchi et al., 2008 [17]; Gong et al., 2007 [56]; Lee et al., 1998 [16]

Based on the PSORT II NNCN score (Table 1), the ROR family RTKs were considered to have good potential for nuclear localization. The NNCN algorithm is used to distinguish the protein tendency to be in either the nucleus or the cytoplasm based on its amino acid composition [39]. We also performed further prediction analysis using other bioinformatics tools (subnuclear [40], ESLpred [41], SubLoc [42], and NpPred [43]). These bioinformatics tools employ a range of different algorithms to perform the prediction: subnuclear is based on the amino acid composition and uses the dipeptide and tripeptide encoding method; ESLpred is based on the physicochemical properties, amino acid composition, and dipeptide composition of proteins; SubLoc is based on the amino acid composition; and NpPred is based on the amino acid, dipeptide, and split composition of proteins. All programs supported the concept that the cytoplasmic region of Ror1 protein has a good nuclear localization potential (Additional file 1). Encouraged by the in silico prediction results, we decided to further examine the cellular expression distribution of the ROR proteins in vivo.

### ROR RTK expression in various cell lines

Because Ror1 is an orphan receptor about which little is known, we first examined the expression pattern of endogenous Ror1 in various human tumor cell lines of different tissue origins. Specific PCR primers were designed to amplify a fragment (CDS: 2169-2311) of the Ror1 transcript (NCBI Reference Sequence: NM_005012). Our screening result identified HT-29 colon cancer cells having the highest Ror1 mRNA expression level (Additional file 2). Further examination of human colorectal cancer samples revealed higher expression of Ror1 in colorectal cancer tissues than in adjacent normal colon tissues (data not shown). In most of the cell lines examined, Ror1 seemed to have a higher or similar expression level compared with Ror2, except in MCF-7 breast cancer cells.

### Determination of nuclear localization domain of the cytoplasmic region of Ror1

**Figure 1:**
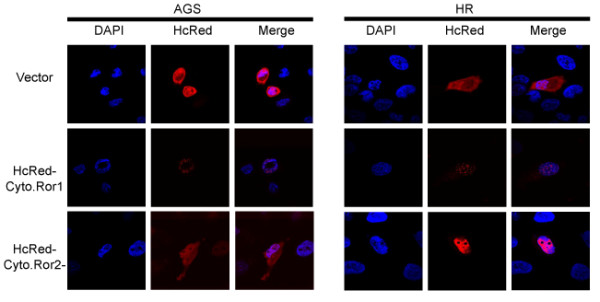
**Ror1/2 mutant with the truncation of the ligand-binding and transmembrane domains are capable of nuclear transport**. AGS and HR cells were transfected with the cytoplasmic part of Ror1/2 whose N-terminal was HcRed-tagged or with a vector control, and then analyzed by fluorescence confocal microscopy. The protein size of HcRed only is about 30 kDa; and the size of the HcRed-tagged Ror1/2 is about 85 kDa.

To explore the nuclear localization potential of the Ror1 cytoplasmic region, we first generated plasmid chimeric fusion constructs (see *Methods*) containing HcRed fluorescent tag protein and the cytoplasmic region of either Ror1 (NCBI Reference Sequence: NP_005003.2) or Ror2 (NCBI Reference Sequence: NP_004551.2) (Figure 1). The localization of the fusion proteins was assessed by detecting the far-red fluorescence emitted by the HcRed fusion protein tags. The HcRed control vector showed an overall homogenous distribution pattern. By contrast, the Ror1 and Ror2 fusion proteins showed a nuclear localization pattern following transfection (Figure 1). The HcRed-Ror1 fusion protein had a unique patched subnuclear localization pattern in cells, whereas the HcRed-Ror2 protein showed a more homogenous nuclear localization pattern. Our data confirmed that the cytoplasmic regions of Ror1 and Ror2 RTKs contain putative nuclear localization signal domains. Because of the good homology between Ror1 and Ror2, we selected the Ror1 gene for subsequent experiments.

Because of the weak but noticeable nuclear expression pattern of HcRed protein in the control vector, we opted to switch to the human influenza hemagglutinin (HA)-tag vector for subsequent studies. To further dissect the domains responsible for the nuclear localization of Ror1, we constructed several Ror1 deletion constructs fused with the HA tag (Additional file 3). Ror1F1 encodes 185 amino acids of the N-terminal Ror1 cytoplasmic region, Ror1F2 encodes 306 amino acids of the C-terminal Ror1 cytoplasmic region, and Ror1F3 and Ror1F4 contained the Ror1 cytoplasmic region with the deletion of the N-terminal 61 residues and 18 residues, respectively. Ror1F3 encodes 424 amino acids, and Ror1F4 encodes 467 amino acids. Following transfection, proper expression of each deletion construct was verified by immunoblot analysis (Additional file 4).

**Figure 2:**
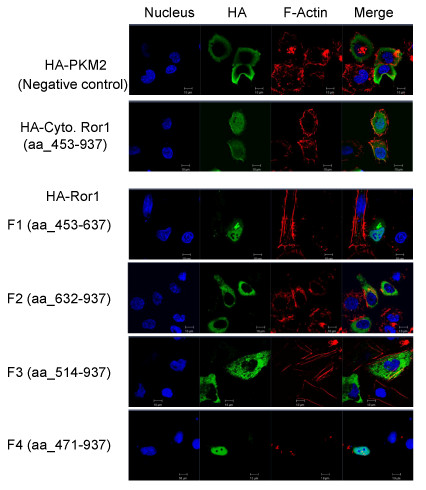
**Identification of the minimal Ror1 domain that plays a critical role in nuclear accumulation**. Confocal microscopic analysis was used to detect subcellular localization of Ror1 fragments. HR cells were transfected for 24 hours with indicated Ror1 fragments and stained with anti-HA monoclonal antibody (green) followed by a fluorescent (FITC) secondary antibody and with phalloidin rhodamine (red). DAPI staining shows the nucleus. PKM2, a known cytoplasmic protein, served as a negative control.

The subcellular protein distributions of these Ror1 deletion constructs were identified using confocal microscopic analysis (Figure 2). As expected, the fusion construct containing the full-length cytoplasmic region of Ror1 localized mainly in the nucleus. The negative control plasmid construct carrying the HA-PKM2 (pyruvate kinase type M2) fusion protein showed only the cytoplasmic distribution pattern. Among the deletion constructs, the Ror1F1 fragment (aa_453-637) localized mainly in the nucleus. By contrast, the Ror1F2 fragment (aa_632-937) localized only in the cytoplasm. Similarly, the Ror1F3 fragment (aa_514-937) also showed a reduced nuclear localization pattern compared with the full cytoplasmic Ror1 construct or Ror1F1 fragment. By contrast, Ror1F4 (aa_471-937) restored the ability to localize in the nucleus. The Ror1F4 fragment was constructed by adding the 471-513 amino acid sequence to the N-terminal of the Ror1F3 fragment. These data suggest that the 471-513 amino acid sequence may be responsible for the observed nuclear localization capability of Ror1 RTK.

**Figure 3:**
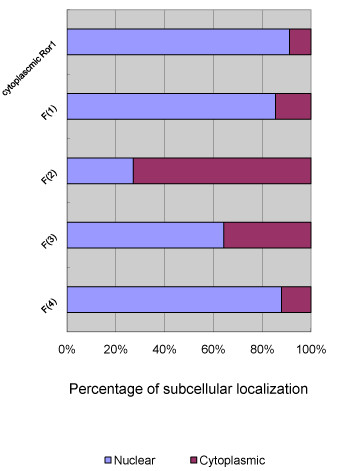
**Quantification of the subcellular distribution ratios of different Ror1 fragments**. The mean percentage of FITC fluorescence in the nuclear or cytoplasmic compartments for each construct was determined by analyzing the FITC intensity from 30 randomly selected cells.

Because Ror1 was reported to interact with the cytoskeleton and because it colocalizes with F-actin during stress fiber formation, we then used rhodamine phalloidin to examine the actin stress fiber formation in these serial deletion constructs. The pericytoplasmic membrane localization of Ror1 fusion proteins and colocalization with F-actin stress fiber were observed in HA-Cyto. Ror1 (aa_453-937) construct. The ratio of nuclear distribution of each Ror1 deletion construct was calculated by quantitatively determining the mean percentage of fluorescein isothiocyanate (FITC) fluorescence intensity from 30 randomly selected cells using the MetaMorph analysis program (Molecular Devices, Inc.) (Figure 3). In brief, our result suggested that the 471-513 amino acid motif located within the juxtamembrane domain is the region responsible for the observed nuclear translocation activity. This region is called Ror1 NLdomain hereafter.

**Figure 4:**
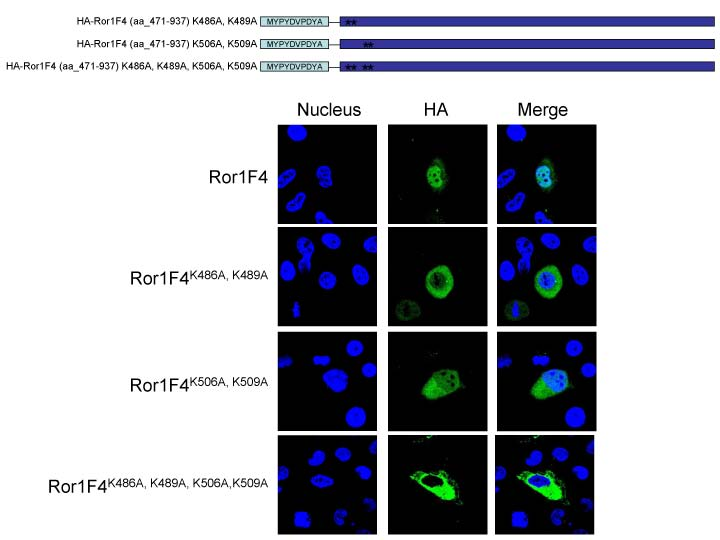
**Site-directed mutagenesis on Ror1 NLdomain unveils one nuclear localization signal**. HR cells were transfected with indicated Ror1 mutants and analyzed using anti-HA monoclonal antibody (green) followed by a fluorescent (FITC) secondary antibody. DAPI staining shows the nucleus.

Because no typical NLS signal peptide sequences are found readily in the Ror1 cytoplasmic region, we examined this region for other possible motifs. Typically, this NLS comprises one or more short sequences of positively charged lysines or arginines. The Ror1 NLdomain does contain a KxxK-16 aa-KxxK bipartite basic charged amino acid pattern. To confirm that this region is truly responsible for the Ror1 nuclear localization, we tried to mutate the four lysines into alanines within this NLdomain region. Residues 486, 489, 506, and 509 were mutated accordingly (illustrated in Additional file 5). The Ror1 nuclear localization was abolished in the mutant constructs examined (Figure 4), suggesting that all four lysine residues are important to the Ror1 nuclear distribution pattern. Our results suggest that the NLdomain located at the juxtamembrane (aa_471-513) region of the Ror1 cytoplasmic domain contributes to the nuclear translocation function.

### Nuclear translocation of Ror1 might be mediated by the RanGTP pathway

**Figure 5:**
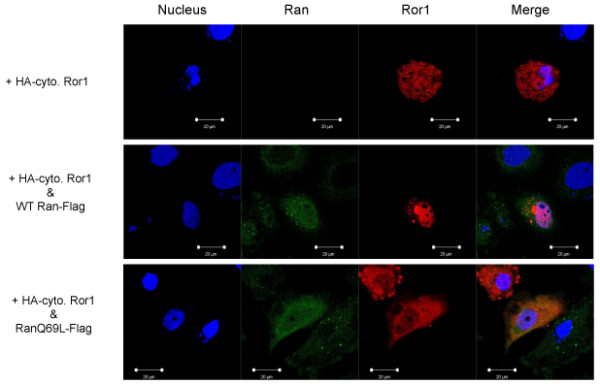
**Confocal microscopy analysis of increased nuclear import of Ror1 by cotransfection with wild-type Ran**. The flag-tagged WT Ran or empty vector control (-) was cotransfected into HR cells with the Ror1 constructs. The protein distribution was analyzed by confocal microscopy.

**Figure 6:**
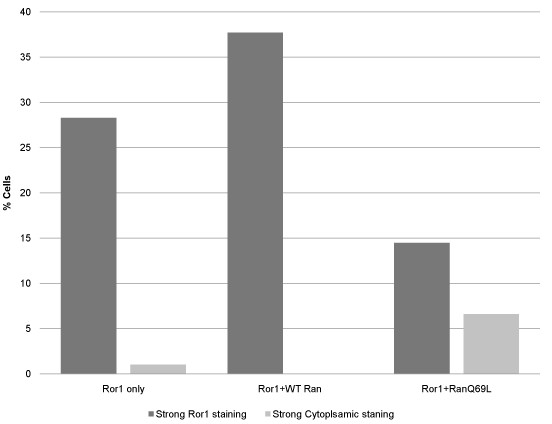
**Quantification of Ror1 localization in transfected cells**. The localization pattern of Ror1 in cells expressing the indicated Ror1 and Ran constructs was scored for 80 cells. The graph shows the percentage of cells with the indicated Ror1 localization patterns.

The nucleotide-bound state of the small GTPase Ran plays a key role in nuclear transport [44]. We examined the possible involvement of such a pathway in the transportation of Ror1 RTK into the nucleus. A dominant-negative mutant of Ran (RanQ69L), which is deficient in GTPase activity, prevents the nuclear import of target proteins [7]. Confocal immunofluorescence analysis showed increased expression of the Ror1 fusion protein in the nucleus of cells cotransfected with wild-type Ran protein but not in cells cotransfected with the control vector. By contrast, the dominant-negative RanQ69L mutant showed reduced nuclear localization of Ror1 fusion protein (Figure 5). Quantification of the Ror1 fusion protein distribution in transfected cells showed that Ran GTPase plays a major role in the nuclear translocation of Ror1 RTK (Figure 6).

## Discussion

In this study, we reported that the cytoplasmic region of the transmembrane RTK Ror1 was able to translocate into the nucleus. This nuclear distribution pattern was similar to that of TrkA RTK. TrkA is a nerve growth factor receptor that shows a predominant nuclear and perinuclear localization resembling the small dense core carrier vesicles [17]. We also observed that cytoplasmic Ror1 formed a number of dense particles near the outer nuclear membrane. Interestingly, Ror1 was originally cloned from the homologous cytoplasmic region of TrkA. The role of the juxtamembrane 471-513 region of Ror1 in determining the nuclear localization identified in this study response to the role of the intracellular portion of TrkA. This conclusion is supported by the evidence showing that nuclear TrkA is recognized only by antibodies against the intracellular region but not by antibodies against the extracellular domain [17].

The juxtamembrane regions of the EGFR family are critical for the nuclear localization of their proteins. The putative NLS in the juxtamembrane domain is conserved among the EGFR family. For example, the putative EGFR NLS sequence is aa_669-RRRHIVRKRTLRR [4], the putative ErbB2 NLS sequence is aa_676-KRRQQKIRKYTMRR [7], the putative ErbB-3 NLS sequence is aa_669-RRQKQNKRAMRR, and the putative ErbB-4 NLS sequence is aa_676-RRKQSIKKKRALRR [10]. We discovered that the 471-513 motif of Ror1 also localizes to the juxtamembrane domain, implying that the nuclear translocation of RTKs might be mechanistically similar. However, there is no traditional nuclear-localized signal sequence such as in the EGFR cases. We found the KxxK-16 aa-KxxK bipartite pattern in this region and showed that it is functional in Ror1 nuclear localization. The 3D-structure modeling of the cytoplasmic part of Ror1 showed that the mapped Ror1 juxtamembrane 471-513 region is exposed at the outer part of the molecule, implying a greater opportunity to be recognized by interacting proteins (Additional file 6).

Nuclear localized RTKs are found to be present as full-length protein (ErbB-1, ErbB-3 and FGFR-1) or proteolytic cytoplasmic fragments (ErbB-4) [1]. In the case of ErbB-4 RTK, it is cleaved by the metalloprotease and gamma-secretase following the binding of its ligand [45]. The cleaved cytoplasmic region of ErbB-4 is then translocated into the nucleus. This process is similar to the one operated in the Notch signaling pathway [46]. In this study, our attempts to detect the endogenous Ror1 expression using rat-anti Ror1 antibody showed a small truncated form of Ror1 (~40 kD) in several cancer cell lines (data not shown). The level of this possible truncated fragment was diminished in subsequent si-Ror1 knockdown experiments. We propose that Ror1 is translocated into the nucleus and that this translocation is mediated by the cleavage-releasing mechanism in addition to the Ran GTPase pathway.

## Conclusions

Our study showed that the cytoplasmic region of Ror1 might be localized in the nucleus in human cancer cell lines. Following the analysis using deletion constructs, the juxtamembrane domain comprising 471-513 residues of Ror1 was determined to be responsible for nuclear translocation. The nuclear localization of Ror1 was enhanced further by cotransfection of Ran wild-type protein but not by the mutant construct.

## Methods

### Cell lines

HR and AGS (human gastric cancer cell line) cells were maintained in Dulbecco's Modified Eagle Medium (DMEM) (12100-038, Invitrogen) plus 10% fetal bovine serum (04-001-1A, Biological Industries) and grown at 37°C in 5% CO_2_. Cultures were passaged when they reached 80% confluence.

### Plasmid construction

pHcRed1-C1_cyto. Ror1 (CDS#1357-2814) vector was constructed by cloning the 1,458 bp cDNA fragment containing the cytoplasmic part coding for the sequence of human Ror1 into the XhoI and KpnI site downstream of the cytomegalovirus (CMV) immediate early promoter of the pHcRed1-C1 plasmid (Clontech). The insert is expressed as fusion with the C-terminus of HcRed1.

pHcRed1-C1_cyto. Ror2 (CDS#1333-2832) vector was constructed by cloning the 1,500 bp cDNA fragment containing the cytoplasmic part coding for the sequence of human Ror2, which was digested from the pGEMT Easy_cyto. Ror2 plasmid into the SalI and SacII site downstream of the CMV immediate early promoter of the pHcRed1-C1 plasmid (Clontech). The insert is expressed as fusion with the C-terminus of HcRed1.

pXJN-HA_cyto. Ror1 (CDS#1357-2814) vector was constructed by cloning the 1,458 bp cDNA fragment containing the cytoplasmic part coding for the sequence of human Ror1 into the HindIII and SmaI site downstream of the CMV promoter of the pXJN-HA plasmid. The insert is expressed as fusion with the C-terminus of the HA tag.

pXJN-HA_Ror1F1 (CDS#1357-1911) vector was constructed by cloning the 555 bp cDNA fragment containing the cytoplasmic part coding for the sequence of human Ror1 into the HindIII and SmaI site downstream of the CMV promoter of the pXJN-HA plasmid. The insert is expressed as fusion with the C-terminus of the HA tag.

pXJN-HA_Ror1F2 (CDS#1894-2814) vector was constructed by cloning the 921 bp cDNA fragment containing the cytoplasmic part coding for the sequence of human Ror1 into the HindIII and SmaI site downstream of the CMV promoter of the pXJN-HA plasmid. The insert is expressed as fusion with the C-terminus of the HA tag.

pXJN-HA_Ror1F3 (CDS#1540-2814) vector was constructed by cloning the 1,275 bp cDNA fragment containing the cytoplasmic part coding for the sequence of human Ror1 into the HindIII and SmaI site downstream of the CMV promoter of the pXJN-HA plasmid. The insert is expressed as fusion with the C-terminus of the HA tag.

pXJN-HA_Ror1F4 (CDS#1411-2814) vector was constructed by cloning the 1,404 bp cDNA fragment containing the cytoplasmic part coding for the sequence of human Ror1 into the HindIII and SmaI site downstream of the CMV promoter of the pXJN-HA plasmid. The insert is expressed as fusion with the C-terminus of the HA tag.

To generate NLS mutants of Ror1, specific alanine mutations were constructed by site-directed mutagenesis using the two-round PCR method [45]. The resulting PCR products were cloned into the pXJN-HA plasmid.

pXJN-HA_Ror1F4 (CDS#1411-2814)^K486A, K489A ^vector has two mutations in the first basic amino acid cluster in the Ror1 NLdomain. The mutations include CDS# 1456AAA→GCA and 1465AAA→GCA. The fragment was constructed by cloning the 1,404 bp cDNA containing the cytoplasmic part coding for the sequence of human Ror1 into the HindIII and SmaI site downstream of the CMV promoter of the pXJN-HA plasmid. The insert is expressed as fusion with the C-terminus of the HA tag.

pXJN-HA_Ror1F4 (CDS#1411-2814)^K506A, K509A ^vector has two mutations in the second basic amino acid cluster in the Ror1 NLdomain. The mutations include CDS# 1516AAG→GCA and 1525AAA→GCA. The fragment was constructed by cloning the 1,404 bp cDNA containing the cytoplasmic part coding for the sequence of human Ror1 into the HindIII and SmaI site downstream of the CMV promoter of the pXJN-HA plasmid. The insert is expressed as fusion with the C-terminus of the HA tag.

pXJN-HA_Ror1F4 (CDS#1411-2814)^K486A, K489A, K506A, K509A ^vector has four mutations in the first and second basic amino acids cluster in the Ror1 NLdomain. The mutations include CDS# 1456AAA→GCA, 1465AAA→GCA, 1516AAG→GCA, and 1525AAA→GCA. The fragment was constructed by cloning the 1,404 bp cDNA containing the cytoplasmic part coding for the sequence of human Ror1 into the HindIII and SmaI site downstream of the CMV promoter of the pXJN-HA plasmid. The insert is expressed as fusion with the C-terminus of the HA tag.

All clones were verified by DNA sequencing and western blotting to confirm the correct sizes of the proteins.

### Plasmid transfection

Expression plasmids were transfected into AGS or HR cells using the Lipofectamine™2000 reagent (11668-500, Invitrogen) as suggested by the manufacturer but with some modifications. Briefly, 18 hours before the experiments AGS or HR cells were plated onto coverslips in six-well culture plates (200,000 cells/well). For the biochemical experiments, cells were plated at a higher density (400,000 cells/well). For each transfection, 2 μg of plasmid DNA suspended in 50 μl of DMEM was combined with 2 μl of Lipofectamine2000 suspended in 50 μl of DMEM and incubated for 20 minutes at room temperature to allow DNA-liposome complexes to form. Meanwhile, the culture medium was changed to serum-free DMEM. The mixture was then added dropwise to the cultures and incubated at 37°C in 5% CO_2 _for 4-6 hours. After incubation, the cultures were changed to DMEM plus 10% fetal bovine serum. The cells were harvested 24 hours after transfection and analyzed.

### Immunocytochemistry

The cells seeded on the coverslips were washed with PBS. AGS or HR cells were fixed for 15 minutes with 3.7% formaldehyde in PBS and then permeabilized in 0.1% Triton X-100 in PBS for 5 minutes. The coverslips were preincubated in 5% bovine serum albumin (BSA) in PBS for 20 minutes at 37°C and exposed to the primary antibodies (diluted in 1% BSA + 0.1% Triton X-100 in PBS) for 1 hr at 37°C. Finally, the cultures were rinsed three times in PBS for 3 minutes and incubated with secondary antibodies for 1 hour at 37°C. After secondary antibodies incubation, the cultures were washed three times in PBS for 10 minutes and then reserved in the mounting medium (Vector). The following primary antibodies were used: HA.11 monoclonal antibody (1:150, MMS-101P, Covance, Emeryville, CA); Anti-HA polyclonal Y-11 antibody (1:100, sc-805, Santa Cruz); and anti-Flag monoclonal M2 antibody (1:100, F3165, Sigma). The following secondary antibodies were used: anti-mouse IgG FITC-conjugated (1:100, sc-2099, Santa Cruz Biotechnology) and anti-rabbit IgG-Rhodamine-conjugated (1:100, sc-2091, Santa Cruz). To detect actin filaments, cells were stained with phalloidin rhodamine (1:100, R415, Invitrogen) for 1 hour at 37°C. As a negative control, one coverslip was incubated without the primary antibody.

### Confocal microscopy and image analysis

Confocal images of transfected cells were acquired using an LSM510 META confocal microscope (Carl Zeiss MicroImaging, Inc.) with an argon laser operating on a Nikon Optiphot upright microscope with an oil immersion 60× 1.4NA objective. Optical sections were acquired at 0.5 mm steps and the XY-pixel size was set at 0.20 mm using LSM510 v3.5 software (Carl Zeiss MicroImaging) and later processed using ZEN2007 v4.5.0.124 (Carl Zeiss MicroImaging). The fluorescence intensity was measured using MetaMorph Offline v7.1.7.0 (Molecular Devices). The intensity of the Ror1 fragment-HA-FITC fluorescence distribution in the nucleus and cytoplasm was estimated by integrating the average fluorescence intensity and areas. The noncellular background was estimated and subtracted from the images before analysis.

### Protein electrophoresis and immunoblotting

Cells were rinsed twice in cold PBS and scraped into PBS buffer, and the pellets were collected in 1.5 mL centrifugation tubes. Cells were lysed with RIPA buffer containing 50 mM Tris-HCl, pH 7.4, 150 mM NaCl, 1% Triton X-100, 1% sodium deoxycholate, 0.1% sodium dodecyl sulfate (SDS), and freshly added protease inhibitor cocktail (11697498001, Roche). Next, the protein concentration was determined using the BioRad protein assay. The protein sample was separated by SDS-polyacrylamide gels and electro-blotted to PVDF membrane. The membrane was blocked with 5% silk milk in TBS containing 0.1% Tween-20 and incubated with the indicated primary and secondary antibodies at room temperature. HA.11 monoclonal antibody was used (1:5000; MMS-101P, Covance). Secondary antibodies conjugated to HRP (1:5000; Amersham Pharmacia Biotech) followed by enhanced chemiluminescence reagents (Western Lightning™ plus-ECL, PerkinElmer, Inc.) were used to detect proteins. In some experiments, blots were stripped in stripping buffer (62.5 mM Tris pH 6.8, 2% SDS, and 100 mM β-mercaptoethanol) at 50°C for 10 minutes, washed with PBST twice for 10 minutes, and then reprobed with a different antibody. Membranes were imaged on LAS-3000 Camera Image Systems (Fujifilm, Tokyo, Japan). Bands were analyzed using Image J v1.36b (NIH, Bethesda, MD).

### Reverse transcription-polymerase chain reaction (RT-PCR)

Total RNA was extracted using TRIzol Reagent (Invitrogen) according to the TRIzol Reagent manufacturer's protocol. Reverse transcription was performed in 20 μl reactions containing 5 μg of sample RNA, 200 U of SuperScript™ III reverse transcriptase, 500 ng of oligo(dT)_15_, 500 μM dNTP mix, 5 mM DTT, and 1X First-strand Buffer (Invitrogen). Tubes were incubated at 50°C for 1 hour and then at 70°C for 15 minutes to terminate the reaction. The reaction products were then subjected to PCR amplification. PCR was performed in 25 μl reactions containing RT product, 0.8 μM forward primer (F), 0.8 μM reverse primer (R), deionized water, 0.2 mM dNTP mixture, 1× PCR buffer (Mg^2+ ^plus), and 0.5 U of TaKaRa rTaq™ DNA polymerase (TaKaRa). The following primer sets were used: Ror1-F4 (5'-CCT CAT GAC AGA GTG CTG GA-3') and Ror1-R2 (5'-TTG TCT GTG TGG TGG CAT TT-3') corresponding to coding sequences 2,169-2,188 and 2,292-2,311 of the Ror1 human sequence, respectively; Ror2-F2 (5'-GGG CAA CCT TTC CAA CTA CA-3') and Ror2-R2 (5'-CGT TGC TCA CAT TGC TCA CT-3') corresponding to coding sequence 2,247-2,266 and 2,331-2,350 of the Ror2 human sequence, respectively; and GAPDH-1F (5'-TGGTATCGTGGAAGGACTCA-3'), GAPDH-2R (5'-AGTGGGTGTCGCTGTTGAAG-3') corresponding to coding sequence 504-874 of the GAPDH human sequence. For the Ror1 and Ror2 primer sets, an initial denaturation time of 5 minutes at 94°C and then 35 cycles (30 seconds at 94°C, 30 seconds at 54°C/56°C, and 30 seconds at 72°C) followed by a final extension time of 10 minutes at 72°C were performed. For the GAPDH primer sets, annealing temperature was set at 54°C. PCR products were separated by electrophoresis on 2% agarose gels in TAE buffer and visualized with ethidium bromide staining.

## Authors' contributions

HCT performed the bioinformatic analysis and Ror1 experiments, and prepared the manuscript. PCL helped with the interpretation of the structure comparison and bioinformatic data. WCL participated in the experimental design, coordination, and drafting of the manuscript. All authors read and approved the final manuscript.

## Supplementary Material

Additional file 1 **Supplementary Table S1**. Prediction of Ror1 subcellular localization by bioinformatic software Click here for file

Additional file 2 **Supplementary Figure S1**. RT-PCR analysis of mRNA expression of Ror1/2 in several human cancer cell lines. RNA extracted from 13 human cancer cell lines and one monkey cell line was used in RT-PCR to detect Ror1/2 gene expression. The relative gene expression was normalized against the gene expression level of GAPDH in the following cells lines. Stomach: AGS, AZ521, and HR; liver: Hep3B, HepG2, and Huh7; lung: H1299; fibroblast: HEL299; cervical: HeLa; blood: HL60; colon: HT29; prostate: LNCap; breast: MCF-7; monkey kidney: Cos-7. Click here for file

Additional file 3 **Supplementary Figure S2**. Illustration of HA-tagged Ror1 fragment constructs. Ror1F1 is the first 1/3 fragment in the Ror1 cytoplasmic part. Ror1F2 is the last 2/3 fragment in the Ror1 cytoplasmic part. Ror1F3 is the fragment of the Ror1 cytoplasmic part with the first 62 amino acids deleted. Ror1F4 is the fragment of the Ror1 cytoplasmic part with the first 19 amino acids deleted. Click here for file

Additional file 4 **Supplementary Figure S3**. Western blot analysis of the expression of HA-tagged Ror1 fragment constructs and HA-tagged PKM2 with anti-HA antibody. Expression of Ror1 recombinant constructs observed by western blot analysis. Detailed construct information is illustrated in supplementary figure 2. Click here for file

Additional file 5 **Supplementary Figure S4**. Schematic representation of the Ror1 NLdomain and the site of the putative NLS. The Ror1 NLdomain localizing in the juxtamembrane region of Ror1 is indicated. The Ror1 NLdomain contains a putative KxxK-16 aa-KxxK bipartite basic charged amino acid pattern. The positions of the four lysine sites are shown in red. Click here for file

Additional file 6 **Supplementary Figure S5**. A theoretical structural model of the cytoplasmic part of Ror1. The 3D structure including the Ror1 NLdomain and intact kinase domain was modeled using FAST Alignment and Search Tool. The dark blue indicates the exposed N-terminal tail. Click here for file
