# Supplementary material for: Nuclear localization of orphan receptor protein kinase (Ror1) is mediated through the juxtamembrane domain
Source: BMC Cell Biol. 2010 Jun 30;11:48. doi: 10.1186/1471-2121-11-48 (PMC2907318; doi:10.1186/1471-2121-11-48)
Supplement: Additional file 1 — Supplementary Table S1. Prediction of Ror1 subcellular localization by bioinformatic software [file 1471-2121-11-48-S1.DOC]

**Supplementary Table 1**. Prediction of Ror1 subcellular localization by bioinformatic software

| Result | Prediction tool | Tool description |
| --- | --- | --- |
| Nuclear | PSORT II [38] | General eukaryotic localization prediction (based on PSORT, iPSORT) |
| Nuclear Lamina | Subnuclear [40] | Predict subnuclear localization by AA module |
| Nuclear Protein | ESLpred [41] | SVM for eukaryotics using Dipeptide composition & PSI-BLAST  Hybrid Approach Based |
| Nuclear | SubLoc [42] | SVM prediction system based on amino acid composition alone |
| Nuclear protein | NpPred [43] | A web server for prediction of nuclear proteins (HMM-based Pfam search + SVM) |
| Cytoplasmic | LOCtarget [59] | Database for structural genomics targets |
