# Supplementary figures and images for: Nuclear localization of orphan receptor protein kinase (Ror1) is mediated through the juxtamembrane domain
Source: BMC Cell Biol. 2010 Jun 30;11:48. doi: 10.1186/1471-2121-11-48 (PMC2907318; doi:10.1186/1471-2121-11-48)

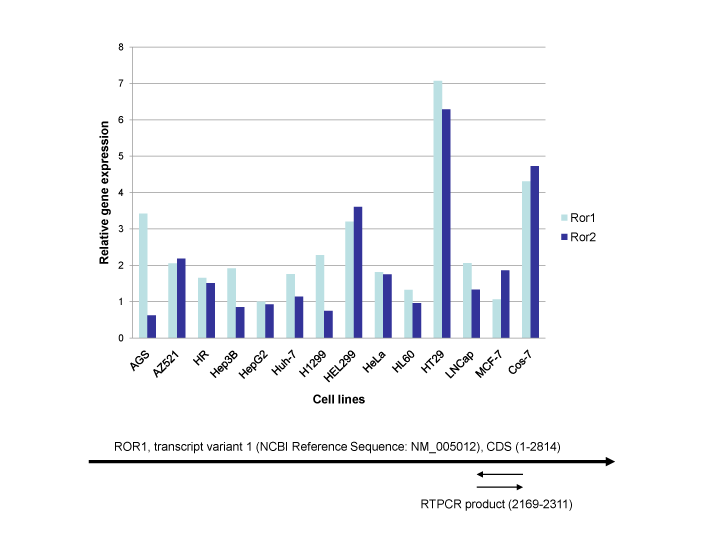

Supplement: Additional file 2 — Supplementary Figure S1. RT-PCR analysis of mRNA expression of Ror1/2 in several human cancer cell lines. RNA extracted from 13 human cancer cell lines and one monkey cell line was used in RT-PCR to detect Ror1/2 gene expression. The relative gene expression was normalized against the gene expression level of GAPDH in the following cells lines. Stomach: AGS, AZ521, and HR; liver: Hep3B, HepG2, and Huh7; lung: H1299; fibroblast: HEL299; cervical: HeLa; blood: HL60; colon: HT29; prostate: LNCap; breast: MCF-7; monkey kidney: Cos-7. [file 1471-2121-11-48-S2.TIFF]

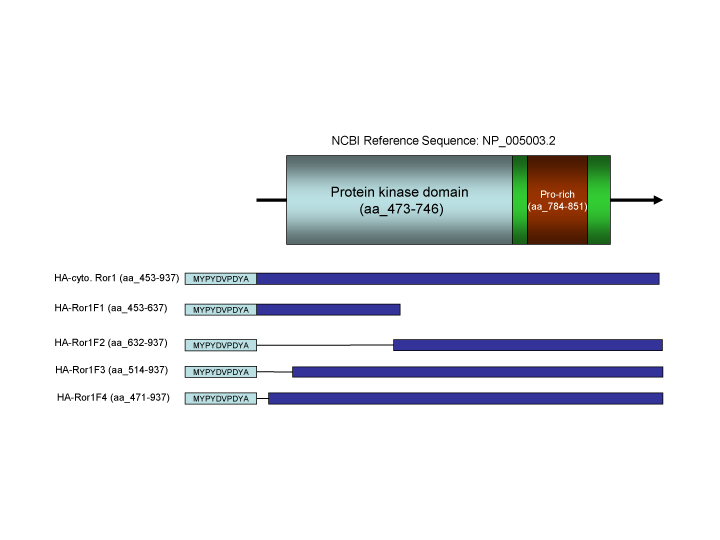

Supplement: Additional file 3 — Supplementary Figure S2. Illustration of HA-tagged Ror1 fragment constructs. Ror1F1 is the first 1/3 fragment in the Ror1 cytoplasmic part. Ror1F2 is the last 2/3 fragment in the Ror1 cytoplasmic part. Ror1F3 is the fragment of the Ror1 cytoplasmic part with the first 62 amino acids deleted. Ror1F4 is the fragment of the Ror1 cytoplasmic part with the first 19 amino acids deleted. [file 1471-2121-11-48-S3.TIFF]

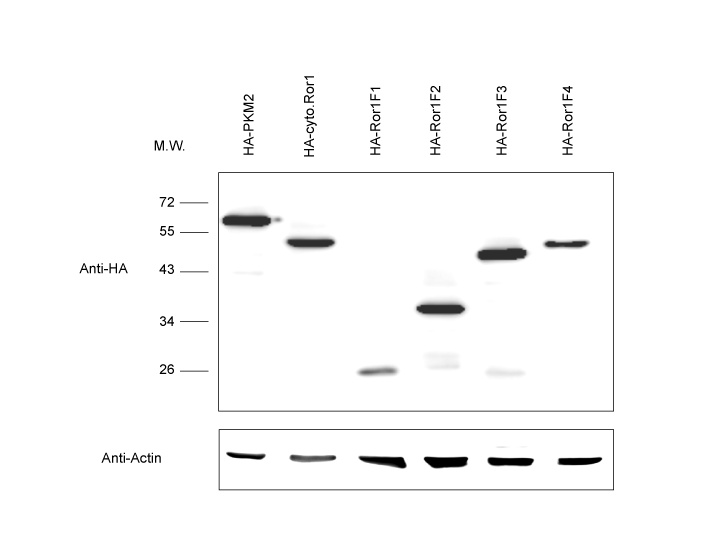

Supplement: Additional file 4 — Supplementary Figure S3. Western blot analysis of the expression of HA-tagged Ror1 fragment constructs and HA-tagged PKM2 with anti-HA antibody. Expression of Ror1 recombinant constructs observed by western blot analysis. Detailed construct information is illustrated in supplementary figure 2. [file 1471-2121-11-48-S4.TIFF]

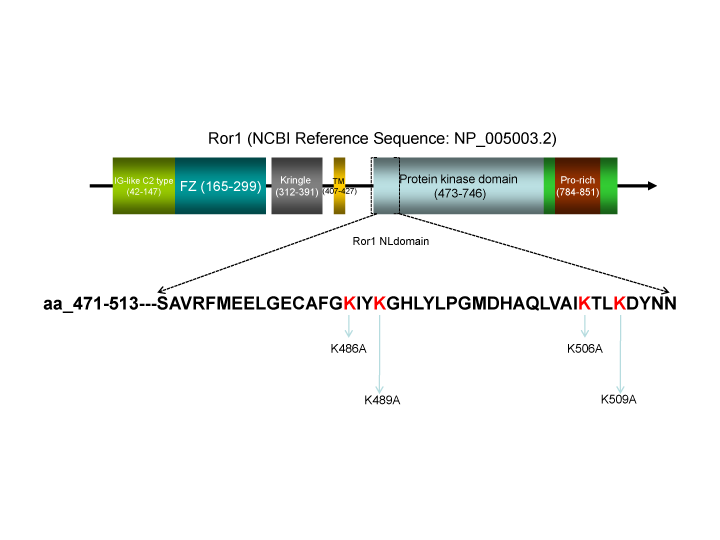

Supplement: Additional file 5 — Supplementary Figure S4. Schematic representation of the Ror1 NLdomain and the site of the putative NLS. The Ror1 NLdomain localizing in the juxtamembrane region of Ror1 is indicated. The Ror1 NLdomain contains a putative KxxK-16 aa-KxxK bipartite basic charged amino acid pattern. The positions of the four lysine sites are shown in red. [file 1471-2121-11-48-S5.TIFF]

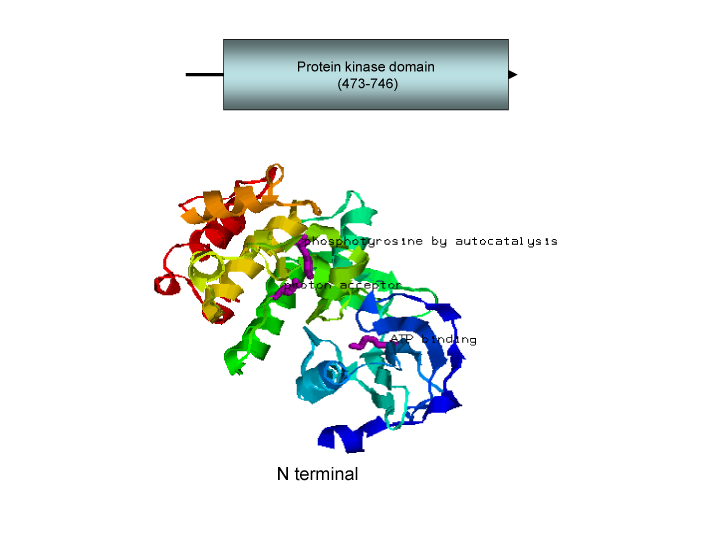

Supplement: Additional file 6 — Supplementary Figure S5. A theoretical structural model of the cytoplasmic part of Ror1. The 3D structure including the Ror1 NLdomain and intact kinase domain was modeled using FAST Alignment and Search Tool. The dark blue indicates the exposed N-terminal tail. [file 1471-2121-11-48-S6.TIFF]
